# One size does not fit all: Participants’ experiences of the selfBACK app to support self-management of low back pain—a qualitative interview study

**DOI:** 10.1186/s12998-022-00452-2

**Published:** 2022-10-03

**Authors:** Malene J. Svendsen, Barbara I. Nicholl, Frances S. Mair, Karen Wood, Charlotte D. N. Rasmussen, Mette J. Stochkendahl

**Affiliations:** 1 Department of Sports Science and Clinical Biomechanics, University of Southern Denmark, Campusvej 55 Odense M, DK-5230 Odense, Denmark; 2 General Practice and Primary Care, Institute of Health and Wellbeing, University of Glasgow, Glasgow, GB UK; 3 The National Research Centre for the Working Environment, Copenhagen, Denmark; 4 Chiropractic Knowledge Hub, Odense, Denmark

**Keywords:** Low back pain, Self-management, Smartphone app, Implementation, Engagement, Digital health, mHealth, Barrier, Facilitators

## Abstract

**Background:**

Low back pain (LBP) is one of the most common reasons for disability globally. Digital interventions are a promising means of supporting people to self-manage LBP, but implementation of digital interventions has been suboptimal. An artificial intelligence-driven app, selfBACK, was developed to support self-management of LBP as an adjunct to usual care. To better understand the process of implementation from a participant perspective, we qualitatively explored factors influencing embedding, integrating, and sustaining engagement with the selfBACK app, and the self-perceived effects, acceptability, and satisfaction with the selfBACK app.

**Methods:**

Using a qualitative interview study and an analytic framework approach underpinned by Normalization Process Theory (NPT), we investigated the experiences of patients who participated in the selfBACK randomized controlled trial (RCT). Interviews focused on the motivation to participate in the RCT, experiences of using the selfBACK app, and views about future intended use and potential of using digital health interventions for self-management of LBP. Participants were purposively sampled to represent diversity in age, sex, and implementation reflected by a proxy measure of number of app-generated self-management plans during the first three months of RCT participation.

**Results:**

Twenty-six participants aged 21–78, eleven females and fifteen men, with two to fourteen self-management plans, were interviewed between August 2019 and April 2020. A broad range of factors influencing implementation of selfBACK within all constructs of NPT were identified. Key facilitating factors were preferences and beliefs favoring self-management, a friendly, motivational, and reassuring supporter, tailoring and personalization, convenience and ease of use, trustworthiness, perceiving benefits, and tracking achievements. Key impeding factors were preferences and beliefs not favoring self-management, functionality issues, suboptimal tailoring and personalization, insufficient time or conflicting life circumstances, not perceiving benefits, and insufficient involvement of health care practitioners. Self-perceived effects on pain and health, behavior/attitude, and gaining useful knowledge varied by participant.

**Conclusions:**

The high prevalence of LBP globally coupled with the advantages of providing help through an app offers opportunities to help countless people. A range of factors should be considered to facilitate implementation of self-management of LBP or similar pain conditions using digital health tools.

**Supplementary Information:**

The online version contains supplementary material available at 10.1186/s12998-022-00452-2.

## Introduction

Low back pain (LBP) is one of the most common reasons for disability globally [1, 2], and the burden has increased over the last three decades [3, 4], accounting for the highest amount of health care service spending in the US in 2016 [5]. Evidence-based self-management tailored to the individual needs and abilities of patients is recommended [6–10] as a first-line treatment strategy for nonspecific LBP. The self-management strategies should include providing patients with information about the condition, and advice to stay physically active including regular exercise sessions [6, 11–14]. A notable barrier to participating in community-based self-management programs is the logistic challenge of accessibility [15–17]. Digitalizing interventions has been suggested as a means to overcome these barriers [18–20], with evidence that digital interventions to support self-management of LBP are effective for improving pain intensity or pain-related disability [21, 22]. However, the uptake of such digital interventions has been suboptimal both in patients with LBP [23] and other patient populations [24, 25].

selfBACK is an artificial intelligence-based smartphone app that aims to facilitate self-management of LBP [26, 27]. The core components of the self-management intervention include education, physical activity, and exercises with behavior change theory ingredients to promote uptake and utilization of the app. Every week, the user is offered a new self-management plan. The plan is tailored to the individual user and based on their interaction with the app and answers to tailoring questions, and by the artificial intelligence-driven system creating new plans based on past successful plans [26, 28, 29]. The effectiveness evaluation of selfBACK showed positive but limited effect on pain-related disability [30]. To date, there is limited literature examining barriers and facilitators to patient uptake and utilization of digital interventions to promote the effective self-management of LBP [23]. Hence, as part of the evaluation of selfBACK, a parallel process evaluation was undertaken to help us better understand the process of implementation from a participant perspective and cast light on how and why participants engaged with the selfBACK app [31].

In this paper, we qualitatively explore the implementation of selfBACK on (i) factors influencing embedding, integrating, and sustaining engagement with the selfBACK app, and (ii) the self-perceived effects, acceptability, and satisfaction with the selfBACK app.

## Methods

### Design

We conducted a parallel process evaluation alongside a randomized controlled trial (RCT), which involved provision of self-management support for LBP through a smartphone app [27, 31] (selfBACK). The process evaluation involved qualitative semi-structured interviews with those participating in the intervention arm of the selfBACK trial. Intervention arm participants had the selfBACK app installed on their smartphone in addition to being offered usual care. The RCT had two areas of recruitment; Trondheim, Norway, and Odense, Denmark, and the app was offered to the participants in their native language (i.e., Norwegian or Danish). Interviews were undertaken with participants from both countries. We followed the COREQ checklist for reporting qualitative studies [32].

### Participants

From August 2019 to April 2020, participants were recruited after they had had access to the selfBACK app for 3 months and after completion of web questionnaires assessing the primary outcome of the RCT. To obtain a study sample with diverse experiences as reflected in a proxy measure for implementation, we undertook purposive sampling to ensure inclusion of participants with different numbers of app-generated self-management plans, and different ages and sex. We considered the number of plans as a proxy measure for implementation. Plans were generated when participants completed weekly tailoring sessions. If participants did not use the app for more than a week, the tailoring session would begin upon the participant’s return to using the app. The minimum number of possible plans was 1 and the maximum was 14 over the 3 months. During the recruitment period, 165 persons became eligible for participation. Of these, 52% (n = 86) had 13 or 14 plans, 30% (n = 50) had 7 to 12 plans, and 18% (n = 29) had less than 7 plans. Drawing on Malterud et al.’s concept of information power [33], e.g. needing fewer because of the specificity of the phenomenon (implementation of selfBACK), and more because of the variation in number of plans and limited experience of the interviewers, a sample of up to 24 participants was deemed appropriate [31]. The data collection stopped, when interviews had been conducted with participants with low (less than 7 plans), moderate (7 to 12 plans) and high (13 or 14 plans) use and data saturation was reached.

The possibility of participating in an interview was included in the information letter and consent form signed by participants at entry to the RCT. Participants were contacted by phone and, if they were interested, a formal invitation was sent by email or text message. One-to-one interviews were conducted in participants’ homes, workplaces, or university offices or via telephone.

### Procedure

A semi-structured topic guide was utilized, focusing on key areas expected to elicit information relevant to aims of the study (Additional file 1). These included motivation to participate in the RCT, previous experience of self-managing LBP, experiences of using the selfBACK app, and views about future intended use and potential of using DHIs for self-management of LBP. The topic guide was pilot tested with three colleagues who had used early versions of the app and with two RCT participants and was considered to work well. Before the start of the interviews, participants were reminded of the content of their signed consent forms. MaJS, a female PhD student (MSc sports science and health), and SAS, a female physiotherapist (MSc PT) employed as a research assistant in selfBACK, conducted all interviews in Denmark and Norway, respectively, and in their respective languages. Before this study, MaJS had no experience with qualitative studies, while SAS had conducted qualitative interviews previously. Interviews were audio-recorded using digital recorders (Olympus Digital Voice Recorder WS-852) and lasted between 18 and 76 min (mean 42 min). The interviewers kept field notes and reflective journals (pre and post interviews). All interviews were transcribed verbatim and imported into NVivo software (QSR International, version 12). MaJS compared all transcripts with the audio files to ensure transcript quality. Participants were offered the opportunity to comment or correct the transcript before analysis.

### Data analysis and theoretical framework

Data were analyzed using a framework approach following the five stages of qualitative analysis described by Ritchie et al. [34]: familiarization, identifying a thematic framework, indexing and sorting, framework summarization, and abstraction and interpretation. Transcripts were coded in their original language. MaJS (who is bilingual in Danish and Norwegian) read each transcript repeatedly for data familiarization and to start an initial thematic coding tree. One full-length transcript translated into English (by SAS, proficient in English at an advanced professional level) was independently coded by MaJS, MeJS (Chiropractor, PhD), BN (Mixed methods expert, PhD), and KW (Social Scientist, MA), and coding was subsequently discussed to ensure consensus on themes and codes of the coding tree. MaJS undertook the coding of the remaining transcripts guided by FSM (Clinical professor of General Practice). When new themes emerged or second opinions were needed, MaJS, MeJS, BN, and KW worked in coding clinics at regular intervals over a 4-month period with smaller fractions of translated transcripts (translated by MaJS, English proficiency at advanced professional level). All members of the author group had been involved in the development of the SelfBACK app [26], in planning the RCT [27], and had had access to use the app prior to the data collection; thus, they had intimate knowledge of the app objectives and functionalities. Reflective journals were kept to monitor the effect of the authors views on the analytic process.

The framework approach was underpinned by Normalization Process Theory (NPT) [35–37]. NPT is an implementation theory that has been widely used, particularly in the digital health sphere to aid understanding of the work people do to embed and sustain service innovations or new technologies in their everyday lives. Embedding a practice is within NPT seen to depend on “a set of ideas about its meaning, uses and utility and socially defined competencies” [35, page 542]. NPT has four constructs: coherence (sense-making work); cognitive participation (engagement work); collection action (operationalization work); and reflexive monitoring (appraisal work). NPT was used to inform the questions of the interview topic guide. Further, the topic guide was informed by the findings of a previous systematic review we conducted on uptake and utilization of digital interventions for the self-management of low back pain, which also used NPT as a guiding framework [23]. In the initial analytic familiarization and coding phase, NPT was not used to allow for the emergence of themes falling outside the NPT framework. Once the data had been summarized and displayed in framework matrices to detect key dimensions of the data [34], it was conceptualized through a NPT-lens which was used to aid understanding of the implementation processes at play. The coding framework and related core constructs of is presented in Table 1. Multiple coding clinics between the author group (MaJS, MeJS, BN, KW, and FSM were organized to discuss abstraction and interpretation of the data through the lens of NPT. Importantly coding clinics were also arranged to ensure openness to identification of themes outside the NPT framework. Original language quotes for the manuscript were translated into English by MaJS.

**Table 1:** Coding framework and related core constructs of Normalization Process Theory, modified from Svendsen et al. [23]

Coding framework	Core constructs of NPT
How people understand and view the benefits versus disbenefits of selfBACK and decide whether it is appropriate for them to use Motivation and willingness to commit to self-management activities	Coherence (Sense-making work; enrolling with / embedding selfBACK): development of an individual and collective understanding of the new intervention when faced with operationalizing it
Willingness to “buy into” selfBACK and whether it is a legitimate means to promote self-management of LBP Issues relating to the support provided to use of selfBACK and level of engagement of HCPs involved with selfBACK	Cognitive Participation (Relational work; engaging with or integrating selfBACK): relational work to build and sustain engagement with a new intervention
Ease of use, accessibility and appropriateness of selfBACK Resources, training, workload and technical support Perceived quality and trustworthiness of selfBACK content and function	Collective Action (Operational work; utilizing and engaging in use of selfBACK): investment of effort and resources to enact the new intervention
How people judge the new selfBACK and the self-monitoring work that accompanied uptake of the selfBACK Ability to match an individual’s needs	Reflexive Monitoring (Appraisal work; maintaining/sustaining engagement with selfBACK): evaluation of the impact of the new intervention on individuals and groups along with any reconfigurations suggested
*Codes falling outside the NPT framework*	
Inherent personal attributes such as personal physical or cognitive abilities that could promote or inhibit use of selfBACK	

*HCP* health care professional; *LBP* low back pain; *NPT* normalization process theory

### Ethical considerations

The selfBACK trial, including the qualitative process evaluation, was registered with ClinicalTrials.gov (NCT03798288) and approved by national ethical committees in Denmark (S-20182000-24) and Norway (2017/923-6) [27]. All participants had signed written consent forms for participation in the trial. At the start of an interview, participants gave verbal re-consent after the interviewer reminded the participant about the voluntary nature of participation, their withdrawal rights, confidentiality issues, and data storage and protection. Participants were informed that interviews would be recorded before arranging the interview. No reimbursement for participation was given.

## Results

**Fig. 1:**
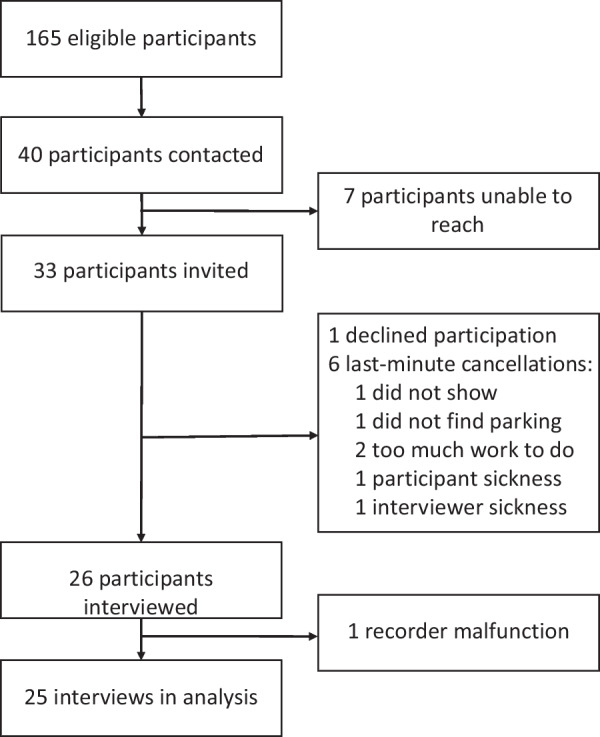
Flow of participants in the study

Figure 1 illustrates the flow of participants. Twenty-six interviews were carried out in participants’ homes, workplaces, or university offices (n = 16) or via telephone (n = 10). Due to recorder malfunction, one telephone interview was excluded from the analysis. The sample had a diverse number of plans and a balanced range of age (mean age 46.1 years, range 21 to 78), sex (females n = 11, 44.0%) and nationality (Danish n = 16, 64%) (Table 2). The study sample was considered to be representative of the entire RCT intervention group in terms of age, sex, and nationality [30].

**Table 2:** Study participants’ demographics and the number of self-management plans during the three months

Participant number	Sex	Age	Country	Number of SM plans generated
1	M	31	Denmark	2
2	M	61	Denmark	2
3	M	21	Denmark	2
4	F	22	Norway	3
5	M	40	Norway	3
6	F	35	Denmark	4
7	M	74	Denmark	5
8	F	57	Denmark	6
9	F	39	Denmark	9
10	M	59	Norway	9
11	F	56	Denmark	9
12	M	23	Norway	10
13	F	57	Norway	11
14	F	58	Denmark	11
15	F	25	Denmark	12
16	M	78	Denmark	12
17	M	47	Norway	12
18	M	63	Denmark	13
19	M	48	Denmark	13
20	M	56	Norway	13
21	F	31	Norway	13
22	M	35	Denmark	14
23	M	29	Denmark	14
24	F	70	Denmark	14
25	M	38	Norway	14

*F* female; *M* male; *SM* self-management

### Findings

**Fig. 2:**
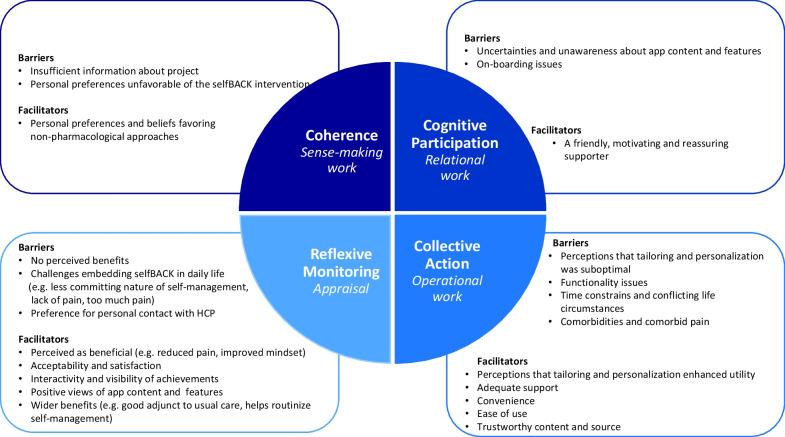
Model of barriers and facilitators influencing implementation of selfBACK. HCP: health care practitioner

Factors facilitating and limiting the implementation of selfBACK emerged in relation to all four constructs of NPT. We identified 14 facilitating and 12 limiting factors influencing embedding, integrating, and sustaining engagement with the selfBACK app (Fig. 2). Below, each construct is explained in detail. A full taxonomy with examples of quotations from participants is shown in Additional File 2. Where quotations are provided, participant numbers refer to Table 2.

#### Level of embedding is associated with personal preferences, beliefs, and level of information (coherence)

Participants’ personal preferences and beliefs at the time of deciding to participate in the intervention affected their sense-making work. Embedding selfBACK was facilitated by having an already active lifestyle and being accustomed to exercising and physical activity, and by recognizing a need for self-management as an adjunct to HCP care:

”I understand that the manual therapy treatments only help if you also do something yourself. So I’ve been doing strength training for the last 5–10 years.” (participant 25 – male, 38).

Conversely, not liking physical activity or believing that group training was superior to exercising on their own limited embedding. Having a positive mindset despite pain, and a strong urge to get better as well as a reluctance to use pain medication or surgery also facilitated the embedding of selfBACK.

“I’m against medications, so I don’t do any and I won’t have any. I don’t believe medications are any good, so instead I had to figure out how to get rid of my low back pain.” (participant 24—female, 70).

Insufficient information about the project, e.g. purpose of the trial or trial duration, were barriers to some participants. Finally, some participants wanted to limit their overall daily screen time, which in turn affected embedding selfBACK.

#### Integration depends on perceived level of support and understanding of app features (cognitive participation)

The relational work of integrating selfBACK in daily life was facilitated by perceiving the app as a friendly and motivating supporter; an in-the-pocket friend that kept reminding them to be physically active, providing information and reassurance, and congratulating accomplishments. Being instructed exactly how to manage their pain, and recognizing advice and exercises already given by their HCP provided reassurance. selfBACK was seen as a casual and non-intrusive helper participants could consult as needed: it was not too pushy, and they could decide for themselves how to accomplish their goals:

“Yes, a bit like an exercise partner who asks ‘hey, shouldn’t we go work out today?’ and then you actually might get going. Where on the other hand, if you were on your own, you might forget about it or take the easy way out.” (participant 22 – male, 35).

Some participants did not explore the entire content of the app and were, thus, unaware of certain supportive app content, which limited their use of the app. For example, a few participants asked for an exercise library (which was part of the toolbox) as they would have liked to be able to perform exercises from previous plans. Another barrier was uncertainty about the purpose of different features of the app, like not understanding why the tailoring session had to be completed weekly. Some ascribed this to the comprehensive onboarding process and the amount of information given when agreeing to participate in the RCT and setting up the app (e.g. signing consent form, randomization, a tour and explanation of the app, and getting the entire selfBACK setup running on the phone [27, 30]).

“My workday ended, I had had a long day, I think I was at the university around 4.30 or 5 pm. Then I had to read something, then I had to sign something, and then I had to use my civil registration system number to log in to the app, and then he had to show me the app, and then there was the wristband, and then I had to look around and see what the app could do, and then… then, ‘here you are, go home and use it’. I mean, that was not enough.” (participant 2 – male, 61).

#### Engagement depends on perceived fit of the app, time consumption, trustworthiness, and functional issues (collective action)

The operational work of routinely engaging with selfBACK was facilitated by the perceived fit of the app content to the individual’s needs. Participants liked that they could customize the content, e.g. by swapping exercises, adjusting their goals, or turning off notifications. The tailoring also meant that many participants felt continuously challenged and interested as exercise difficulty levels and step goals progressed. Participants appreciated the variation of content, weekly updates, and the balanced focus on what was needed, as this participant explained:

“It [updates] was great because then new exercises appeared. And they impacted some different things, I felt. That was good.” (participant 3 – male, 21).

However, when tailoring and personalization were perceived as irrelevant or mismatched for the individual user, engagement was limited. For some, the tailoring sessions were perceived as a nuisance as they could not be bypassed, occurred too frequently, or the questions did not adequately cover the participants’ situations. Others felt the content was repetitive or their plans too ambitious and the goals unrealistic. The app’s focus on exercises and steps was also perceived as irrelevant by participants who preferred biking, swimming, or skiing.

Participants liked that selfBACK was accessible at anytime from anywhere, which meant they saved both time and money by not having to pay for a gym membership or spend time on transportation. Furthermore, participants reported that following the plans required minimal effort as they were broken down into manageable goals and attainable time slots:

“It’s just 20 min. You can spare that in the evening. That’s a… a nice feeling of doing at least something productive. You’re able to allocate 20 min per day or at least a couple of times per week.” (participant 22 – male, 35).

Ease of use (including exercises not requiring equipment and animated, easy-to-grasp real-time videos), notifications, and visual prompts (e.g. seeing the selfBACK logo on phone screen) facilitated engagement. Trustworthiness of selfBACK further facilitated engagement. Participants described the professionalism and evidence-base as reassuring, which made them trust that following the advice from selfBACK would not worsen their LBP. This was further aided as the invitation to participate came from their trusted HCP. Furthermore, support from the research team and talking with friends and family about selfBACK supported engagement with the app.

Barriers to integrating selfBACK activities into daily life included insufficient time as participants had to set aside time for it even when they were busy or had other commitments, or conflicting life circumstances like moving, starting school, or being away on holiday. Comorbidities (e.g. diabetes/neuropathy or fractured bones) and comorbid pain also limited some participants in engaging in selfBACK activities, as one participant explained:

“I’m so tormented by my knee, if I have to lie down to perform some of the exercises, I can hardly get back up. I have to roll onto the other foot and find a piece of furniture to pull myself up by because my knee is so weak. So the exercises for my low back pain, I’m not able to perform them as long as my knee is in such a bad condition.” (participant 10 – male, 59).

Many participants described technical hiccups, such as faulty step count synchronization (from the step-detecting wristband to the selfBACK app), login issues, or finding the screen size too small to properly see the videos. Although a few were discouraged by these technical issues and asked for more technical support, the majority of participants felt these issues did not significantly limit their engagement with the app. Such issues were believed to be normal for technologies. A few participants even described that a daily routine of fixing faulty step synchronization to achieve daily step goals increased engagement. Additional functionality issues limiting engagement included discomfort wearing the wristband and not understanding all of the app content when not speaking the language of the app fluently.

#### Perceived effects, acceptability, satisfaction, and sustained engagement and self-management (reflexive monitoring)

Whether or not participants sustained their engagement with selfBACK was dependent on their perception of the effect and benefit of use. Experiencing a positive effect on pain and health, behavior or attitude, or gaining useful knowledge contributed to sustained engagement just like trusting that using selfBACK would prevent relapses of LBP. On the contrary, participants who had not experienced positive effects of using the app or who had experienced an increase in pain did not sustain their engagement with the app.

Participants experienced effects from using selfBACK in several ways. Their primary focus was on pain intensity or disability level, and many participants experienced improvements in these. Multiple participants reported a recovery from LBP, and described going back to normal life, including attending work, being able to pick up previous sports, like running, or no longer having to avoid pain evoking positions. One participant, who had visited his HCP regularly every month for several years, even forgot his appointments as he had improved considerably:

“It’s been so effective that I actually forgot my visit to the physiotherapist […] I thought, that’s a really great sign. I hadn’t experienced that in several years; if 6 weeks went by instead of 4, then I experienced a significant impairment of my back. So now, just within this half-year or 4 months, I’ve been in this project, I’ve rescheduled. Now we try 6–7 weeks between visits.” (participant 19 – male, 48).

Several participants described how sustaining engagement with selfBACK had improved their general health. Participants reported improved fitness levels, weight loss, improved strength, balance and flexibility, heightened energy levels, and improved sleep cycles. Behavioral and attitudinal effects were described by both participants who had and had not experienced effects on pain outcomes. Initiation of exercising habits, engagement in more and regular physical activity, or small changes like parking further away, or standing up doing office work was reported by some. Others explained how their mindset had changed—feeling more in control of the pain, having a more positive mindset and a better experience of living with LBP. Some described being more conscious about physical activity levels and the necessity of being physically active, as this participant explained:

“I’m not feeling the same kind of pain anymore. I don’t feel sorry for myself in the same way I used to […] it has meant that I’m more aware that my own effort has a large effect on how my back will feel going forward.” (participant 18 – male, 63).

Lastly, many participants described gaining new knowledge about LBP and self-management; knowing now what to do about it. Interactivity and visibility of achievements, for example, the step tracking, rewards, and accomplishment progress, further facilitated sustained engagement, as these participants expressed:

”Well, you’re able to follow exactly how you’re doing in reaching your goal. Compared to just thinking to yourself ‘I have to walk this much today’ then you’re able to follow how far you are from achieving that goal, in percentages. I think that’s positive!” (participant 9 – female, 39).

Some participants described how they struggled to sustain engagement with selfBACK due to the lack of a fixed time point for doing selfBACK activities and having to rely on their own motivation, as these participants explained:

“The things you have to do on your own at home, that’s more bothersome, because the other things [group training], I have to leave [the house] for that. […] I also have an exercise bike at home, it’s been years since I used it last! And that’s the thing about being on your own, it’s a struggle. (participant 16 – male, 78).

During the three months intervention period, the participants described becoming familiarized with the exercises and self-management. As a result, increasingly over time, they failed to log their exercises in the app and limited their use of it. Many participants described forgetting to use the app when LBP decreased, or only performing exercises when pain flared up. Conversely, too much pain also limited engagement:

“I was hard up, I mean I couldn’t… I live in a new first-floor apartment and the car is parked just outside, and even walking the few steps down and out to the car was an overcoming for me. I was very passive and could barely walk.” (participant 7 – male, 74).

A handful of participants requested more interactions with their HCP to discuss e.g. correct performance or alterations of exercises shown in the app. Although selfBACK was never intended to replace the HCP, using the app as an adjunct did not make sense to these participants:

“It hasn’t been good enough for me that it was purely an app. I mean, I missed being followed up.” (participant 2 – male, 61).

Ultimately, selfBACK relied on participants committing to LBP self-management, which these participants did not feel confident enough to do on their own. Additionally, experiencing conflicting advice between the app and the HCP was a barrier to sustained engagement.

Appraisal work was facilitated by features of the app. Participants described selfBACK content as ‘just right’; the combination of content was exactly what they needed to manage their LBP. Repetition of content aided comprehension, especially for the educational messages, as one participant explained:

“I actually think it works really well. Because I think the persistent reminder of getting you to understand that it’s important you keep doing something despite pain works really great in the app.” (participant 11 – female, 56).

Participants benefitted from the tailoring session as it allowed them to reflect on changes in their pain, something they would not have considered without prompting. Furthermore, for some participants, receiving self-management advice in written form, rather than during a (verbal) HCP consultation, was beneficial. Recognizing the wider benefits of selfBACK positively affected participants’ sustained engagement: the novelty of managing LBP with support from an app, the potential socioeconomic benefits of having larger populations use the selfBACK app, and having it as an adjunct to usual care in a primary care setting.

Generally, participants were pleased with selfBACK and would recommend it to others. They liked having selfBACK as an adjunct to usual care as several participants pointed out; they had not lost anything agreeing to participate in the RCT—it was a win–win situation for them. Most participants, therefore, did not have any specific expectations of selfBACK other than generally wanting help for self-managing their LBP. The participants who were less satisfied with selfBACK seemed to be those who would have liked integration of the app in their usual HCP care or solely wanted to attend their HCP. Participants gave specific recommendations for improvements to the selfBACK app (Table 3).

**Table 3:** Recommendations from participants for added features to improve the selfBACK app

Option to self-regulate time points for notifications e.g. prompt for physical activity exactly when the participant thought it was needed
Option to see one’s weekly progress in pain symptoms from the answers to the tailoring session questionnaire
Option to “star mark” favorite exercises
Option to watch exercises performed from different angles to enhance understanding of correct performance
Guidance on tweaking exercises rather than fully replacing exercises
Option to rule out all exercises that required for example weight on knees or wrists

#### Data outside the coding framework

A small proportion of data fell outside our coding framework. The personal attribute of being or not being competitive facilitated or limited implementation, respectively. Similarly, some participants were motivated by reaching goals and getting rewards, whereas others were not. Implementation was also facilitated by feeling committed to the research project and/or the research team.

## Discussion

We have explored factors influencing how people with LBP embedded, integrated, and sustained engagement with the selfBACK app, and how they perceived effects, acceptability, and satisfaction with the app. Self-perceived effects on pain and health, behavior/attitude, and gaining useful knowledge varied by participant. Participants generally expressed satisfaction with the selfBACK app and saw it as an acceptable adjunct to usual care. We identified a broad range of factors influencing the use of selfBACK within all four constructs of the NPT. Our findings suggest that engagement with the app was facilitated when participants had preferences and beliefs favoring self-management, found the app to be a friendly, motivating and reassuring supporter, recognized the app as tailored and personalized to their needs, convenient and easy to use, and trustworthy. Participants also positively appraised the app when they experienced positive effects on pain and their thinking about pain. Conversely, participants who did not buy into the self-management concept, who found the app difficult to use, did not feel the personalization met their needs, had insufficient time or conflicting life circumstances, lacked positive effects, or sought more interaction with an HCP were less likely to positively appraise and engage with the app.

We found that participants with pre-existing positive self-management beliefs more often positively appraised the content of the app and used it more. Previous studies have similarly shown that users of eHealth interventions for chronic pain [14] and mental health programs [38] who can see the benefits of self-management programs have increased engagement. The interview participants implemented selfBACK variably, but 52% (n = 86/165) of the eligible participants had 13 or 14 plans, which was reflected in this study’s sample. In the interviews, these “high-end” users reported using the app daily. They used selfBACK as they experienced an effect from using it continuously and were satisfied with the app, and even though they experienced barriers, they found ways to overcome them. Previous research has pointed to an important relationship between people’s desire and motivation to self-manage pain [39] and their goals and values. By engaging with an eHealth self-management program, users are provided with a tool to maintain a sense of control over life [40].

Key facilitators of app utilization found in our study and other studies include ease of use and convenience for participants [23, 41, 42] and users feeling supported and reassured [23, 41]. We identified a group of participants with ten to twelve plans who engaged initially, then discontinued use around two months from baseline, as they improved so much that they no longer felt a need for the app. This use pattern relates to the idea of “effective engagement” where reaching the intended outcome terminates engagement [43]. Our finding also speaks to the dynamics of self-management support and shows how self-management needs are changeable over time [44]. On the other hand, setting and reaching achievable and measurable goals are well-known behavior change techniques that ultimately support self-management. We observed that those who perceived benefits, e.g. either directly on pain or function, behavior, mindset, or new knowledge, sustained their engagement with the app. This aligns with existing research on self-management of chronic pain [45].

Several participants in our study described how the ‘just right’ combination of content was exactly what they needed to manage their LBP and how the educational parts made them change their thinking about pain. According to Dwarswaard et al., several factors influence how people build self-confidence and become empowered [44], and ultimately, stay engaged with a self-management program. The need for professional psychosocial support is key to building self-confidence and becoming empowered [14, 23, 41, 42, 46], and could explain why some of the selfBACK participants wanted more follow-up from their HCP. Typically, these participants did not experience an effect on pain and did not report much satisfaction, had few plans generated, and reported many barriers that limited their engagement, but mostly felt that their HCP was superior to using the app. We tested the selfBACK app as an adjunct to usual care, but the two were not integrated. Our results indicate that for some, selfBACK alone was probably not enough to get them engaged in self-management, and although it was never intended to replace the HCP but rather work as an adjunct to usual care, self-management could be improved if integrated into usual care and followed up by the HCP. Ultimately, even though most participants perceived positive benefits and were satisfied with selfBACK, it is not a ‘one size fits all’ – despite the sophisticated artificial intelligence to tailor the app to the participants. For some patients with LBP, an app to support self-management may not be suitable as it does not align with their preferences and feeling of autonomy [40].

The participants reported a range of technical problems. Similar functionality issues have also been shown to be barriers to use in other studies [23, 41, 42, 47]. Interestingly, most of our participants reported the technical issues as minor, something that was normal or expected of all apps and e-based technologies. This differs from previous findings of a systematic review [23] where poor IT usability was an important barrier. The literature base for the systematic review dated from 2014 and before. It seems that the use of apps for self-management is more natural for patients today than when the studies in the systematic review were conducted, which supports the acceptability of self-management app use.

### Methodological considerations

This study benefitted from researcher triangulation [48] with quadruple coding and repeated coding clinics, as well as continuous collaboration and discussion about interpretations. A rich dataset with varying user profiles in terms of number of plans allowed us to understand how participants had actually engaged with the selfBACK app. However, several limitations must be noted. We included no follow-up interview, which could have cast light on long-term engagement rather than intended sustainment. Furthermore, no data on socioeconomic status or ethnicity was included to describe participants, but the study sample was predominately Caucasian middle-to-upper class. On top of that, our participants were able to commit both time and energy to a research project requiring them to engage in self-management of their LBP *and* to participate in an interview study about their experiences. Although efforts in ensuring a balanced diversity in number of self-management plans aimed to capture participants who had been less satisfied and/or discontinued use for some reason, the study sample may be assumed to consist of quite motivated participants. Recruiting participants from an RCT means that we have not reached participants for whom using an app for self-management would never come into question and their voices are yet to be heard.

Participants’ level of implementation of selfBACK did not follow a linear process from the initial interest when their HCP invited them to participate, to finding it easy to use, to perceiving benefits and therefore sustaining engagement. Instead, participants described the process as iterative and circular, e.g., thinking that generally, apps for supporting health behavior were clever with a potential to help countless people with LBP in the present or future, to recognizing the convenience of equipment-free home exercises, to finding the selfBACK app friendly, motivational and reassuring, and then to perceiving effect on their pain, which matches the constructs of NPT and their interconnection. It is noteworthy that very little data fell outside the NPT coding framework and any that did, consisted of inherent personal attributes such as being/not being competitive or feeling a strong commitment to serving research. This suggests that NPT was a useful theoretical framework to apply in the analytic process to understand participants’ experiences of LBP and use of the selfBACK app. Many other implementation frameworks and theories exist [49] that might have covered the data falling outside NPT or picked up other aspects of implementation than NPT, but not necessarily led to the same level of depth of understanding of the barriers and facilitators to selfBACK implementation.

### Implications for practice and future research

Based on the findings of this study, practice and future research recommendations include the following:

- As most participants were satisfied and perceived benefits from implementing selfBACK, our findings should support clinicians in exploring patients’ interest in using selfBACK or similar apps for self-management of LBP as an adjunct to usual care.
- Self-management of chronic pain, including LBP, is a key treatment strategy, but something many patients struggle to achieve. Although self-management of LBP with selfBACK was supposed to work as an adjunct to usual care, this was achieved by some but for others, not integrating HCP follow-up limited implementation. HCPs remain key players in supporting self-management behavior [50], and we suggest incorporating solutions that allow users and HCPs to share the app content and monitor progress. Having the opportunity to discuss app content and features with the HCP should be incorporated in future, similar apps. Building on experiences from the selfBACK RCT, a clinician dashboard to facilitate co-decision making is currently in development [51].
- In this study, we have reported on implementation of the selfBACK app from a participant perspective only, but as key players in supporting self-management, it is vital we also aim to understand the perspective of HCPs. Future research should explore HCPs’ barriers for use of the selfBACK and similar apps, as well as means and incentives to overcome identified barriers. In continuation, clinical practice barriers relating to provision of digital interventions, and organizational and systemic healthcare system barriers should be targeted.
- Digital health interventions have been suggested as essential for overcoming future challenges of limited resources in the healthcare sector. Despite the potential value, there is only limited research to support cost-effectiveness. We recommend future research focus on the economic impact of selfBACK and other digital interventions on patients’ healthcare utilization and need for social services or workers compensation.

## Conclusion

We identified a number of key factors involved in embedding, integrating and sustaining engagement with the selfBACK app. Participants were generally satisfied with the selfBACK app and many experienced positive effects. The high prevalence of LBP globally coupled with the advantages of providing help through an app offers opportunities to help countless people dealing with LBP in daily managing their pain. A range of factors should be considered to facilitate implementation of self-management of LBP or similar pain conditions. These findings should help inform development of future pain/LBP self-management apps.

## Supplementary Information

**Additional file 1.** General interview guide and Normalization Process Theory domains.

**Additional file 2.** Full taxonomy with exemplar quotes. Participant numbers refer to Table 2.

## Data Availability

The data is available (in original language) from the corresponding author upon reasonable request.

## Abbreviations

COREQ: Consolidated criteria for reporting qualitative research
DHI: Digital health intervention
HCP: Health care practitioner
LBP: Low back pain
NPT: Normalization process theory
