# Supplementary material for: One size does not fit all: Participants’ experiences of the selfBACK app to support self-management of low back pain—a qualitative interview study
Source: Chiropr Man Therap. 2022 Oct 3;30:41. doi: 10.1186/s12998-022-00452-2 (PMC9531397; doi:10.1186/s12998-022-00452-2)
Supplement: Supplementary file 1 — Additional file 1. General interview guide and Normalization Process Theory domains. [file 12998_2022_452_MOESM1_ESM.docx]

Additional file 1. **General interview guide and Normalization Process Theory domains.**

| **Topic** | **Objective** | **Questions** | **NPT domain** |
| --- | --- | --- | --- |
| Background | Warm up, start conversation and get to know informant | Please tell me a little bit about yourself and your everyday life |  |
| Motivation | Learn about what informants thought selfBACK would be and how and with what it could help them. | How did you become part of the selfBACK project?  What made you say yes to participate in selfBACK?   - *Follow*-*up*: What was it (the kind of help) you thought you needed? | Do participants ‘buy into’ the selfBACK app concept? (CP)  Do participants understand the purpose of the selfBACK app? (C)  Do they think it is a good idea? (c)  Do participants have confidence in the app? (CA)  How do participants judge its value? (RM) |
| Self-management before selfBACK | Insight in how informants managed their LBP before selfBACK, maybe reflexion about necessity of self-management | How did you manage your low back pain before selfBACK? |  |
| Experience of using the app | Actually using the app and integrating it into daily life:  What has been/is barriers and facilitators for using the app; effect of using it; appraisal. | What makes you use the app?  How do you use the app?   - *Follow-up:* Was that also how you used it in the beginning?   Which part(s) of the app content have you valued the most?  What has meant for you to use the app?  Would you recommend others to use the app? | Is it easy to use? (CA)  Can they fit app use into their daily lives? (CA)  Are there contextual issues that help/hinder use? (CA)  Do participants have confidence in the app? (CA)  What are the perceived benefits of the app use? (RM) |
| Future use | What is hindering the informant from integrating the app into daily life?  Do they value the app enough to sustain engagement? | Do you imagine (keep on) using the app in the future?     - *Follow-up:* What could make you want to do it? | How does it need to be altered, if at all? (RM) |
| General opinion/ experience | More generally about self-management and digital health interventions, not specifically related to selfBACK | What do you think about this ‘self-management’ that selfBACK is aiming to support?  What do you think about using apps designed to help you get/feel better? | Do participants ‘buy into’ the selfBACK app concept? (CP) |

C=Coherence; CP= Cognitive participation; CA= Collective action; RM=Reflective monitoring
