# Supplementary material for: One size does not fit all: Participants’ experiences of the selfBACK app to support self-management of low back pain—a qualitative interview study
Source: Chiropr Man Therap. 2022 Oct 3;30:41. doi: 10.1186/s12998-022-00452-2 (PMC9531397; doi:10.1186/s12998-022-00452-2)
Supplement: Supplementary file 2 — Additional file 2. Full taxonomy with exemplar quotes. Participant numbers refer to Table 2. [file 12998_2022_452_MOESM2_ESM.docx]

Additional File 2: Full taxonomy with exemplar quotes. Participant numbers refer to Table 2.

| FACTORS ENABLING IMPLEMENTATION | Exemplar quotes | FACTORS PROHIBITING IMPLEMENTATION | Exemplar quotes | NPT CONSTRUCTS |
| --- | --- | --- | --- | --- |
| **Personal preferences and beliefs favoring non-pharmacological approaches**   - Liking physical activity and having an active lifestyle prior to intervention - Positive mindset despite pain - Strong urge to get better - Accepting and preferring self-management - Reluctance against pain medication and surgery | “I’m against medications, so I don’t do any and I won’t have any. I don’t believe medications are any good, so instead I had to figure out how to get rid of my low back pain.” (part 24 - female, 70)  ”I understand that the manual therapy treatments only help if you also do something yourself. So I’ve been doing strength training for the last 5-10 years.” (participant 25 – male, 38)  “In my spare time, I like to be very physically active, probably a bit more than what’s actually necessary. I play a lot of soccer, two days per week on average, I try to practice a couple of runs each week. I like to be fit.” (participant 25 – male, 38)  "If I don't figure out how to manage my LBP now, I'll feel like this for the rest of my life and that's unbearable." (part 24 - female, 70)  “There were talks about surgery but that’s not of any interest at all because you risk getting worse and I’m not willing to risk that.” (part 20 – male, 56)  ”Its’ because… you can say I use this daily and it’s what keeps me on top of things, keeping me at it. Because, without a doubt, I’m doing best when I’m staying active” (part. 14 – female, 58)  ”Often, especially when talking about back pain - that’s my understanding and I do have some experience with it – that when you’re staying active and keeping yourself busy, then you’re doing great. It’s when you become sedentary, that’s when the joints stiff.” (part. 5 – male, 40)  ”I’ve been doing some rounds at my chiropractor’s, and the back pain improved, but I think… so when the doctor mentioned this project and told me about what it was, I got the impression that it was a test study about help to self-help, and that sounded really great!” (part. 5 – male, 40)  ”I thought that’s a great idea. Because I do know that if I’m physically active, I feel and get better. And I’m also fully aware that it’s no good slouching in a couch or an armchair.” (part. 8 – female, 57) | **Insufficient information about project**   - Uncertainty of purpose - Uncertainty of trial period   **Personal preferences unfavorable of the selfBACK intervention**   - Not liking physical activity - Preferring group training - Limiting screen time | “No, let me tell you. I’ve previously done research projects myself, so I guess I have a somewhat different approach towards this. I think I would have liked to be more informed about the purpose, informed about what the overall purpose was.” (part. 7 – male, 74)  “I actually thought that it was three months and then that was it.” (part. 16 – male, 78)  “I’m not someone who’s the happiest about doing things like that. I find those kind of exercises incredibly boring. I mean, everything about exercising is boring. I’ve done a lot throughout my life time […] but I’ve just never felt that working out was particularly interesting, no matter which sport I tried.” (part. 7 – male, 74)  “I’m no workout enthusiast, I don’t like to.. I work out because I have to, not because I want to.” (part. 10 – male, 59)  “Well, it’s the team spirit feeling that makes you show up and do your exercises. I bike a little at first, getting warmed up, and then we do the exercises, maybe an hour to 75 minutes. That, I wouldn’t have accomplished at home.” (part. 7 – male, 74)  “I try not to sit with it way too much... not be like I have to have my phone in my hand when I’m on the bus and stuff like that.. I try to put it aside.” (part. 21 – female, 31) | Coherence |
| **A friendly, motivating and reassuring supporter**   - Motivational and supportive app - Casual and non-intrusive help - Providing reassurance | “Yes, a bit like an exercise partner who asks ‘hey, shouldn’t we go work out today?’ and then you actually might get going. Where on the other hand, if you were on your own, you might forget about it or take the easy way out.” (part. 22 – male, 35)  ”Then I thought I had someone who looked after me and cared for me, right in my pocket at all times.” (part. 10 – male, 59)  “Well, it’s really pleasantly casual. You decide yourself how much or how little, and when and how you’ll do it.” (part. 18 – male, 63)  “It’s really great with the exercise videos, you’re able to double check that you’re doing everything correct.” (part. 21 – female, 31)  “[…] do it the right way, not exaggerate the pain. And have a push… getting it done right but also a focus on doing it more often.” (part. 12 – male, 23)  “Yes, those videos were just brilliant. I could see someone else perform it correctly and then I could copy that. It’s not like when you get a picture because with that it can be a little difficult understanding how you’re supposed to do it.” (part. 12 – male, 23)  “But when I got this app, then it got much easier to focus on it every day because I didn’t just sit there clueless of what I had to do. I felt like ‘I know what I have to do with these exercises’, and then it got much easier to get them done every day.” (part. 12 – male, 23)  ”I thought that now I have someone who looks after me, right there next to me at all times. There’s a lot I don’t get, but the things about steps and such which is the most important… So I’ve gotten a sort of “attendant” who looks after me all the time, who follows me around the clock.” (part. 10 – male, 59) | **Uncertainties and unawareness about app content and features**   - Unaware of content - Uncertainty of purpose of content   **On-boarding issues**   - Too much information - App installation issues - Poor timing of set-up meeting | “I’ve not really been sure if it’s deliberate, if these exercises are specifically chosen for me. It of course can’t examine me, but if there’s been a reason to why these exact exercises were chosen for me…” (part. 22 – male, 35)  “You’re able to adjust it [step goal] down. I’ve never tried to increase it. I don’t think you’re able to do that.” (part. 18 – male, 63)  “I don’t know if it’s… is it intentionally that it keeps asking me ‘How’s your low back pain this week and has it affected your everyday life?’ all those sorts of things. Or is it something that ends?” (part. 15 – female, 25)  “My workday ended, I had had a long day, I think I was at the university around 4.30 or 5 pm. Then I had to read something, then I had to sign something, and then I had to use my civil registration system number to log in to the app, and then he had to show me the app, and then there was the wristband, and then I had to look around and see what the app could do, and then… then, ‘here you are, go home and use it’. I mean, that was not enough. To me, that was not enough.” (part. 2 – male, 61)  ”There was a lot of trouble getting the app installed on my phone, both myself and… I can’t remember his name, on the fourth floor of [clinic]? [interviewer suggests name of possible research assistant] yes, maybe, yes. We had quite some trouble. It took awhile before it worked.” (part. 7 – male, 74)  ”I feel strongly about this two-way-communication, think it’s so great, you know. So if she, the researcher I met, had had time to talk to me in addition, much more around just installing the app and sending me out the door again… But she did say “Do you have any questions?” so it’s not all her fault, it’s mine too. But it’s easier to see someone and then it’s that someone who should present this brand new world for you, she’s the one who should provide all the information in my opinion. Maybe it’s a tad foul against her, but I say this to you now, it’s not a critique of her, it’s just now’s my opportunity to say what I think if we’re supposed to keep on with this.” (part. 10 – male, 59)  “I might not have been so good at reading these, it’s a bit like manuals for things we buy.” (part. 5 – male, 40) | Cognitive Participation |
| **Perceptions that tailoring and personalization enhanced utility**   - Tailoring intervention to increase personal fit - Narrow focus - Progression and challenges - Customization - Variability of content - Updating of content/plans   **Adequate support**   - Assistance from research team - Discussing use with friends and family   **Convenience**   - Saving time and money - No time restriction - Attainable plans   **Ease of use**   - Visual reminders and notifications - Task-based reminder - Animated, real-time guidance of exercises - Simplicity of exercises not requiring equipment   **Trustworthy content and source**   - Professionalism and evidence-base - Trustworthy source of referral | “It [updates] was great because then new exercises appeared. And they impacted some different things, I felt. That was good.” (part. 3 – male, 21)  “I do feel the, for example for the exercises, they get more and more difficult. And sometimes you think ‘oh, I simply can’t do this’ but then you also become a little stubborn and think ‘yes, I will be able to do this’. And then you do it! Even if the first time you think this actually hurts a bit more than expected but then when you’ve done it.. and many of the exercises are 3x10 and it might only be the first round that’s achy. Then the next 2x10 don’t. And then afterwards you’re feeling really great!” (part. 14 – female, 58)  “You can have good times and bad times so I think it’s super this… that when you get your weekly plan, the app suggests a step goal and then it’s possible to adjust it yourself. And I’ve adjusted it both up and down throughout the period… depending on how well you think you’re doing with your back.” (part. 14 – female, 58)  ”I think the variation’s been quite good… and that’s good. It’s good because then you’re sort of forced to keep up and go in and take a look, so you don’t just pushes ahead with the same things [exercises], and I think the body definitely benefits from that, that the exercises vary.” (part. 18 – male, 63)  “Yes, once a week you get a… and then it’s updated. And then you might get some new exercises or something. I think that’s quite effective!” (part. 22 – male, 35)  ”I do notice that for instance with the exercises, they get more and more difficult. And sometimes you think ‘hey, I’m not able to do that’ but then you get a little stubborn and think ‘sure, I can do that’ and then you do it anyway! Even though maybe the first time you think ‘oh, that maybe hurts a bit more than what I expected’, right? But then you’ve done it. And many of these exercises are like 3x10 and maybe it’s only the first [set] that’s hurtful. Then the next 2x10 aren’t. And then afterwards you’re feeling super!” (part. 14 – female, 58)  “The first thing you did when logging in to the app was to decide for yourself how much you wanted, or how much time you wanted to spend on activities or on exercises and.. so you got quite free hands to it and that was nice and easy to understand.” (part. 12 – male, 23)  “It was nice to be followed-up without having to go to the physiotherapist” (part. 4 – female, 22)  “It’s just 20 minutes. You can spare that in the evening. That’s a… a nice feeling of doing at least *something* productive. You’re able to allocate 20 minutes per day or at least a couple of times per week.” (part. 22 – male, 35)  “You decide yourself how much or how little and when and how […] and again, this thing about being able to do the exercises whenever you want. And it doesn’t require anything other than floor space and a stool. Sometimes a chair.” (part. 18 – male, 63)  “It’s nice not having to say ‘alright, I’m in pain, now I have to buy these pills to lessen it’ or ‘I HAVE to go see a chiropractor to… you know, it’s 350 DKKR. [50$] or something like that just to see her once, and that’s not an unreasonable price, that’s just what it costs depending on your insurance… so, it’s really nice not having to think about financials.” (part. 15 – female, 25)  “It’s a great advantage that you can do it at home and when you’re free instead of having to go from work and spend an hour, and then ywo days later you have to go back again and spend an hour more.” (part. 5 – male, 40)  “You don’t need to wait for available appointments at your doctor’s.” (part. 4 – female, 22)  “It’s only 20 minutes, you’re able to spare that in the evening. So yeah, that’s a good… a feeling of at least doing something productive.” (part. 22 – male, 35)  “Yes, so then when you log in to the app and you get the overview of the exercises… it’s easy to see those four or five exercises and how much time it required to do them. And then press on them and watch how you’re supposed to perform them, the exercises. I mean, that’s fairly easy and you don’t spend, don’t need to spend so much time to accomplish what you need to do.” (part. 19 – male, 48)  “I got the invitation from my chiropractor and I know a little about the foundation of that education and what they’re doing and I trust her 100%, I do. And I also know that NTNU is serious, that you also possess the scientific knowledge, so yes, as an ordinary person I have no problem trusting you and the therapist who advised me to join.” (part. 25 – male, 38)  “It’s reassuring that it’s professionals who have created this app because it’s easy to find various fitness apps but they’re much more, how’d say it, advertisement like than actual evidence-based.” (part. 6 – female, 35) | **Perceptions that tailoring and personalization was suboptimal**   - Repetition of content - Unachievable goals - App activities too narrowly focused - Tailoring session not fully relating to participant   **Functionality issues**   - Technical challenges - Language issues - Comfort issues with wristband - Missing technical assistance from research team   **Time constrains and conflicting life circumstances**  **Comorbidities and comorbid pain** | “They [educational messages] say a lot about taking responsibility for your back injury, or not your back injury, your back pain, it says a lot about that. Then I think ‘Yes, I know. I’ve read it for the eighth time already’” (part. 8 – female, 57)  “If you’ve tried it, or will try it, that every week you have to answer how you’re doing on a scale, how does that look? Because what’s 1 and what’s 10? How do you estimate that when you’re dealing with pain? I don’t believe in that. I mean, I don’t think it makes sense.” (part. 11 – female, 56)  “And, I would have liked that it matched the watch. It was annoying that it only came halfway up, that I had only reached 5500 steps when I had taken 8000. And then it just didn’t work.” (part. 8 – female, 57)  “And then this weekly reminder of tailoring and adjusting step goal appears, but that’s bloody difficult when you [step synch.] won’t cooperate.” (part. 18 – male, 63)  “And then it would be really great if I could have it on my iPad, and that’s just because then it gets enlarged and it’s easier.” (part. 8 – female, 57)  “That bumpy underside of the watch has been unpleasant because I move my wrists around in all directions, so it’s like it’s pushing down on the wrist many times a day. So after just two days I almost had a mark.” (part. 6 – female, 35)  “It’s just that you run around at work all day and your head gets tired, every part of the body is tired […]” (part. 8 – female, 57)  “I work mostly late evening/night, and then they have that unhealthy policy in the firm that you should work with as short as possible transfers, which means sometimes there’s only 11 hours and 15 minutes until you have to start again. And when I have to start and finish in one city and go home to another one, then […] yes, also because it doesn’t always match with the train schedule so I have to take the car back and forth. And you HAVE to have time for sleeping as well.” (part. 18 – male, 63)  “I’m so tormented by my knee, if I have to lie down to perform some of the exercises, I hardly can’t get back up. I have to roll onto the other foot and find a piece of furniture to pull myself up by because my knee is so weak. So the exercises for my low back pain, I’m not able to perform them as long as my knee is in such a bad condition.” (part. 10 – male, 59) | Collective Action |
| **Perceived as beneficial (e.g. reduced pain/improved mindset)**   - Effect on pain and health - Attitudinal and behavioral changes - New knowledge - Prevention of LBP relapse   **Acceptability and satisfaction**  **Interactivity and visibility of achievements**   - Step tracking - Goals and rewards - Achievement progress   **Positive views of app content and features**   - ‘Just right’ content (Appropriateness of content) - Comprehension aided by repetition - Self-reflection on status aided by tailoring session - Written advice trumps verbal   **Wider benefits (e.g. good adjunct to usual care/helps routinize self-management)**   - Recognizing potential socioeconomic effects - Novelty of pain management support - Adjunct to usual care - Reconfiguration (SB becomes superfluous for SM) | “It’s been so effective that I actually forgot my visit to the physiotherapist […] then I thought, that’s a really great sign. I hadn’t experienced that in several years; if 6 weeks went by instead of 4, I experienced a significant impairment of my back. So now, just within this half-year or 4 months, I’ve been in this project, I’ve rescheduled. Now we try 6-7 weeks between visits.” (participant 19 – male, 48)  “I’m not feeling the same kind of pain anymore. I don’t feel sorry for myself in the same way I used to before. […] it has meant that I’m more aware that my own effort has a large effect on how my back will feel going forward.” (participant 18 – male, 63)  “It’s great to get the reminder that, for instance, that thing about physical activity, that there’s a connection and sometimes you should avoid aiming too high, it’s better to build up gradually, and that it’s normal to experience some pain immediately after… but yes, you’re reminded… and exactly that is great, it’s often the same messages again and again but we need that kind of repetition sometimes.” (participant 21 – female, 31)  “I actually think it works really well. Because I think the persistent reminder of getting you to understand that it’s important you keep doing something despite pain works really great in the app.” (participant 11 – female, 56)  “No really, in all seriousness, I think it’s an amazing tool, absolutely. I think people who have similar problems and are willing to use this will get great help. So absolutely something that should be brought forward and made available, I have to say that.” (part. 5 – male, 40)  “Given that I’m noticing how much I’ve actually been walking today, how active I’ve been, when I did my exercises last.. Because back when I just had it on a piece of paper I thought ‘it’s only two days since last time’ and then you check and it was actually eight days ago. (participant 6 – female, 35)  ”Well, you’re able to follow exactly how you’re doing in reaching your goal. Compared to just thinking to yourself ‘I have to walk this much today’ then you’re able to follow how  far you are from achieving that goal,  in percentages. I think that’s positive!” (participant 9 – female, 39)  ”No, I think… the really nice thing is this with the mindset, I think… you easily resign and become sick and tired when you’re constantly in pain.” (part. 10 – male, 59)  “I’m gonna miss the apple, the small texts everyday, I think they’re so good. They probably make me aware of things, I think. Conscious about even if it hurts, it’s probably not broken or harmful.” (part. 10 – male, 59)  “Yes, I think I’ve established a greater awareness about the importance of just doing something.” (part. 11 – female, 56)  “But I actually think the step tracking is what’s most effective, because I keep an eye at ‘oh, did I accomplish that – then I better also remember my exercises in the evening’ or something like that.” (part. 22 – male, 35)  “It’s something DIFFERENT having it visualized that only took 100 steps one day […] I mean, a day where you just stayed in bed. I’ve had periods where I’ve almost laid in bed for a week and isolated myself in a room somewhere. So this thing about having something telling you ‘this does not look good, this step count, could you consider…’. And it’s not because, I do know that thing about the 10.000 steps being an arbitrary number chosen for some marketing add and all that, but it’s a bummer knowing that those 10.000 steps might have benefitted you and you only accomplished 12 to 100 today. Great reminder that you weren’t physically active!” (part. 15 – female, 25)  ”It gives me something, I don’t know, I’m the kind of person who loves to-do lists so when I’ve ticked something off my to-do list I just feel really pleased because then there’s one thing less to do. And contrary, when it tells me ‘now you completed 33% of today’s goal’ well that just gives me something too… a wish getting up at 100%. So it provides me with a goal and it gives some attainable goals compared to ‘You have to walk 10k today’. (part. 11- female, 39)  “So I’ve done it this way, during the day, just by wearing this [wristband], I check my steps. I do that regularly, see how much I’ve been walking and then make sure to get up at level before I get, maybe before I get home from work.” (part. 19 – male, 48)  “No, I’ve been writing in an exercise… yes, I normally make a plan for my workout, then I do my exercises as a part of this.” (part. 4 – female, 22)  ”right now I only have 3 exercises in my plan and I have no trouble remembering those.” (part. 23 – male, 29)  “Of course, it’s also a socioeconomic issue that’s really good, maybe the queue to the general practitioner becomes shorter, sick leave reduces.” (part. 5 – male, 40) | **No perceived benefits**   - No effect on pain or health - Pain increase from self-management   **Challenges embedding selfBACK in daily life (e.g. less committing nature of self-management, lack of pain, too much pain)**   - No fixed time - Less committing than group training - Pain as a prompt - Forgetting to log exercises after completion   **Preference for personal contact with HCP**   - HCP not replaceable by app - App and HCP advice conflicting - Wanting follow-up from HCP | “Three things happen, which becomes decisive for me. First, it doesn’t have the effect on my pain it should have to keep me motivated. It keeps hurting. That’s tiresome.” (part. 11 – female, 56)  “Some of these exercises I’ve gotten more back pain from doing. I’ve… I’ve swapped them from time to time but then you feel like you have to try them anyways but then…” (part. 20 – male, 56)  “I was hard up, I mean I couldn’t… I live in a new first-floor apartment and the car is parked just outside, and even walking the few steps down and out to the car was an overcoming for me. I was very passive and could barely walk.” (participant 7 – male, 74)  “[…] it was easier when I attended the group training class with the other, by the physiotherapist, and you’re forced, or not *forced*, but you’re feeling guilty if you don’t do it, and then one time I was feeling sick and then the other were like ‘Oh, you’ve been away, haven’t you?’… I mean, you get some sort of, also because it was a small group, we were only four people, so you get a sense of duty if you fail to show up.” (part. 9 – female, 39)  “The things you have to do on your own at home, that’s more bothersome, because the other things [group training], I have to leave for that. […] I also have an exercise bike at home, it’s been years since I used it last! And that’s the thing about being on your own, it’s a struggle. (participant 16 – male, 78)  “It hasn’t been good enough for me that it was purely an app. I mean, I missed being followed up.” (participant 2 – male, 61)  “And that’s what I wanted to tell you, it’s definitely the greatest limitation that it’s not a human being.” (part. 11 – female, 56) | Reflexive Monitoring |
| **Codes falling outside NPT** | | | | |
| - Personal attributes (e.g. competitive ) - Level of commitment to research | “I don’t know if it’s because I’m quite competitive […] but I’ve really felt like if I hit the couch at night and only had achieved 8000 steps, I’d get up and gone for a walk to get those steps in and hit the 10.000 steps. Then I got the gold star.” (part. 19 – male, 48)  “Yes, I’m a competitive person and in a way I feel like I have to win in this app.” (part. 25 – male, 38)  “Also because you want to be a bit conscientious towards the project.” (part. 15 – female, 25)  “You push yourself a bit extra, having someone watching over you. And I know, I don’t think someone’s sitting at NTNU following my every move, I don’t think that, but you have that… And I’ve said this, I’ve been pursued, I’ve been pursued since August, I say.” (part. 10 – male, 59)  “Well, now I’ve volunteered for this study, now I have to deliver something for this study.” (part. 22 – male, 35)  “But I promised to be part of this, so even if I haven’t been using the app lately, I’m not breaking a promise.” (part. 7 – male, 74) | - Personal attributes (e.g uncompetitive) | “I do see, I do see a lot of people who probably, who are motivated by… but I’m extremely little competitive.” (part. |  |
